# Compounds from African Medicinal Plants with Activities Against Selected Parasitic Diseases: Schistosomiasis, Trypanosomiasis and Leishmaniasis

**DOI:** 10.1007/s13659-018-0165-y

**Published:** 2018-05-09

**Authors:** Conrad V. Simoben, Fidele Ntie-Kang, Sergi H. Akone, Wolfgang Sippl

**Affiliations:** 1 Institute of Pharmacy, Martin-Luther University of Halle-Wittenberg, Wolfgang-Langenbeck-Str. 4, 06120 Halle (Saale), Germany; 2 Department of Chemistry, Faculty of Science, University of Buea, P.O. Box 63, Buea, 00237 Cameroon; 3 Institute of Pharmaceutical Biology and Biotechnology, Heinrich-Heine-University, Universitaetsstrasse1, Geb. 26.23, Duesseldorf, 40225 Germany; 4 Department of Chemistry, Faculty of Science, University of Douala, PO Box 24157, Douala, 00237 Cameroon

**Keywords:** African medicinal plants, Leishmaniasis, Natural products, Parasitic diseases, Schistosomiasis, Trypanosomiasis

## Abstract

**Abstract:**

Parasitic diseases continue to represent a threat on a global scale, particularly among the poorest countries in the world. This is particularly because of the absence of vaccines, and in some cases, resistance against available drugs, currently being used for their treatment. In this review emphasis is laid on natural products and scaffolds from African medicinal plants (AMPs) for lead drug discovery and possible further development of drugs for the treatment of parasitic diseases. In the discussion, emphasis has been laid on alkaloids, terpenoids, quinones, flavonoids and narrower compound classes of compounds with micromolar range activities against *Schistosoma*, *Trypanosoma* and *Leishmania* species. In each subparagraph, emphasis is laid on the compound subclasses with most promising in vitro and/or in vivo activities of plant extracts and isolated compounds. Suggestions for future drug development from African medicinal plants have also been provided. This review covering 167 references, including 82 compounds, provides information published within two decades (1997–2017).

**Graphical Abstract:**

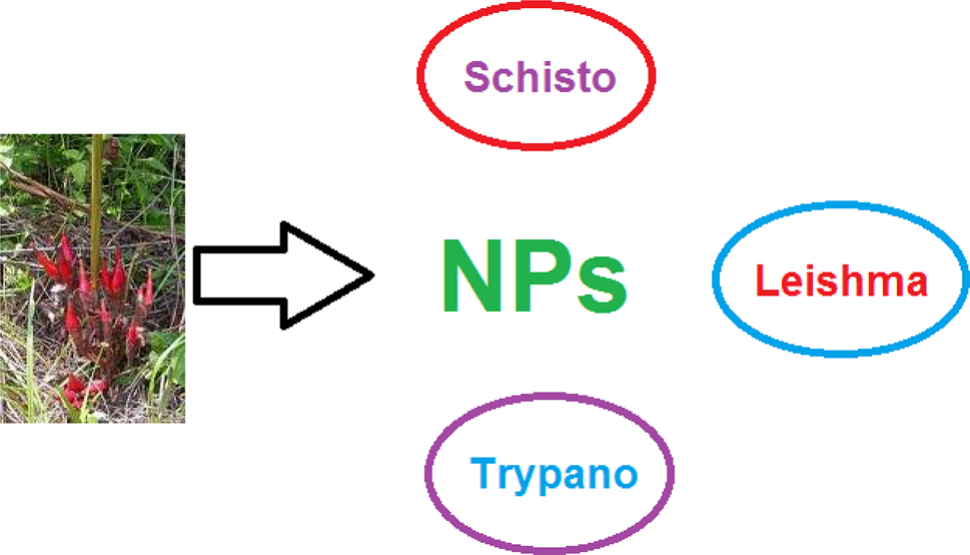

## Introduction

**Fig. 1:**
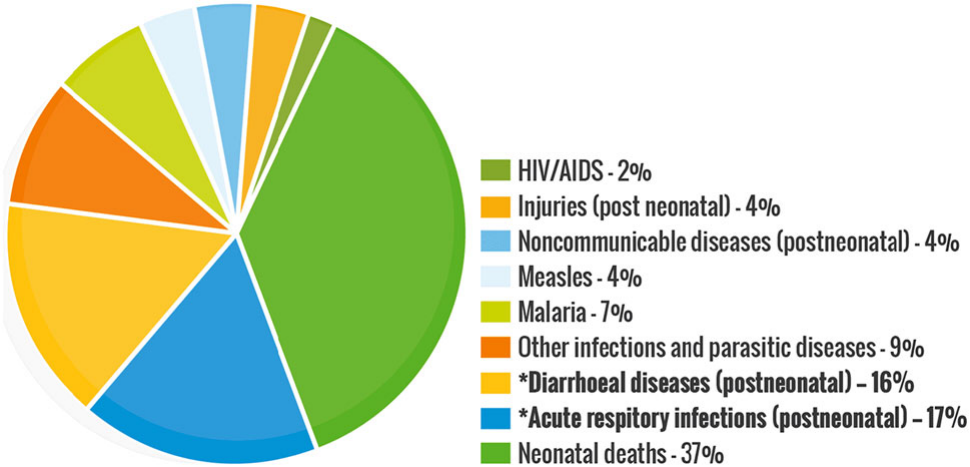
Global statistics for disease burdens in 2017

Parasites are considered as organisms that obtain their food by eating other organisms or their products in nature. These diseases continue to be a cause of considerable morbidity and mortality globally [1–4], including Trypanosomiasis (African sleeping sickness and Chagas disease) [5–7], Leishmaniasis [8] and Schistosomiasis [9, 10]. They threaten almost one-third of the world’s population, the most numerous incidents being recorded in over 100 tropical and developing countries and territories, Fig. 1 [11–13]. The African region recorded the most death-related cases, especially amongst infants below the age of 5 and pregnant women. Schistosomiasis, caused by parasites of the *Schistosoma* genus are responsible for about 200 million sickness cases and about 280,000 death-related incidents annually worldwide [9, 10, 14]. Only one drug (praziquantel) has been proven to be effective in the treatment of human Schistosomiasis, with no vaccine available or in development so far [15–21]. Serious concerns about drug selectivity and resistance were raised in 2013 when over 30 million people were treated in Sub-Saharan Africa [20]. Moreover, observed resistance and reduced efficiency of praziquantel in laboratory strains have prompted the search for alternative therapeutic strategies [20–27].

Trypanosomiasis, which represents several diseases caused by parasites of the genus *Trypanosoma*, is also of interest [5, 27–29]. This disease, which is much arguably the most important disease of man and domesticated animals, accounts for over 8 million reported annual cases globally, especially in the tropical regions of Latin America and Africa [30, 31]. Besides, great socioeconomic effects on the endemic areas by this disease are forecast if inadequate attention (both at the communal, national, and international levels) is not given [7, 29, 32–34]. Leishmaniasis is caused by parasites of the *Leishmania* type, which is also transmitted by certain types of sandflies [35, 36]. The diseases are reported by the WHO to be responsible for about 1 million new cases leading to approximately 30,000 deaths annually on a global scale. The major cause is linked to environmental changes and affects mainly the very poor populations [37, 38]. These three diseases represent a real burden to the lives of millions of persons and their domesticated animals. The trio is capable of inflicting long-term disability and social stigmatisation, which can ultimately lead to a highly unproductive population and eventually result in economic loss and the slowdown of a country’s development.

With the absence of any vaccine targeting any parasites and resistance against the already existing anti-parasitic drugs, research efforts have been employed and encouraged towards the search for new, cheaper, potent and effective drugs to treat these diseases. Medicinal plants represent a potential source of new drugs. This is because natural products (NPs) from organisms such as animals, fungi and the higher plants have been known to be good sources of pharmacologically active compounds against several ailments, including parasitic infections. Moreover, NPs are believed to have significant advantages as lead molecules over synthetic molecules [39–49]. The criteria for choosing a particular natural product for studies are either based on the pre-existing knowledge on the traditional use of the source species in therapy (ethnobotanical knowledge) or the search for structurally related molecules with known pharmacologically active agents from chemical databases [49–54]. The African continent is highly diverse ethnobotanically. This might explain why about 80% of the population tends to rely on medicinal plants as a primary source of healthcare [55–67]. It is our goal to provide evidence of the efficacy and potency of plants used in traditional medicine against parasitic infections. The systematic documentation of the plant-based chemical constituents of African traditional medicine and attempting to using in silico procedures to investigate their modes of action are ongoing efforts [44–46, 52, 53], particularly on the isolated compounds from African medicinal plants (AMPs) with evaluated in vitro and/or in vivo activities against Trypanosomiasis [68–74], Schistosomiasis, Leishmaniasis [72–74] and other parasitic diseases [4]. However, the most recent review dates about 3 years back and was focused only on plants collected from Nigeria. Thus, an updated review that covers the entire continent for these three parasitic diseases is required now. The information presented herein was retrieved by searching literature from major international journals on natural products and medicinal chemistry, alongside available M.Sc. and Ph.D. theses and online databases [54, 75]. The information gathered is discussed under the main compound classes, as presented below and summarised in Tables 1, 2, and 3.

**Table 1:** Bioactive alkaloids from African flora with potential for antitrypanosomal and antileischmanial drug discovery

Compound number	Compound class/subclass	Part of plant studied	Species name	Plant family	Place of collection	Used traditionally/locally	Reported activity on/against	References
**1**–**5**	Alkaloid/Naphthylisoquinoline	Leaves	*Ancistrocladus tanzaniensis*	Asteraceae (Compositae)	Uzungwa Mountains, Tanzania	Different species of *Ancistrocladus* are used as a diuretic; also for the treatment of malaria, dysentery, elephantiasis, febrile and phlogistic.	Trypanosomiasis and leishmanosomiasis	[85, 86]
**6**, **7**		Leaves, stem bark and roots	*Ancistrocladus ealaensis*	Asteraceae (Compositae)	Eala, Democratic Republic of Congo		Trypanosomiasis and leishmanosomiasis	[87]
**8**–**11**		Stem and root bark	*Ancistrocladus congolensis*	Asteraceae (Compositae)	Yandja-Rive, Democratic Republic of Congo		Trypanosomiasis	[88]
**12**–**14**		Leaves	*Ancistrocladus species*	Asteraceae (Compositae)	Ikela, Democratic Republic of Congo		Trypanosomiasis and leishmanosomiasis	[89]
**15**		Roots	*Ancistrocladus likoko*	Asteraceae (Compositae)	Yangambi, Democratic Republic of Congo		Trypanosomiasis and leishmanosomiasis	[90]
**16**		Roots	*Dioncophyllum thollonii*	Dioncophyllaceae	Rabi Kounga, Gabon	For treatment of malaria, Leishmaniasis, dysentery and elephantiasis	Trypanosomiasis and leishmanosomiasis	[91]
**17**, **18**	Alkaloid/Aporphine	Aerial parts	*Cassytha filiformis*	Lauraceae	Sèmè, Ouémé, Benin	To treat cancer, African Trypanosomiasis and other diseases	Trypanosomiasis (IC_50_ = 10.29 and 17.60 μM, respectively)	[92]
**19**–**28**	Alkaloid/Quinoline	Roots	*Waltheria indica*	Malvaceae	Inder, Niger	To treat cough, fever, external haemorrhage, dysentery, toothache, malaria, eye drop	Trypanosomiasis (IC_50_ for 26 = 3.1 μM)	[93]
**29**–**33**	Alkaloid/Indoles and others	Stem bark	*Polyalthia suaveolens*	Annonaceae	Yaoundé, Cameroon	To treat rheumatic pains	Trypanosomiasis (IC_50_ for 31–0.5 μM)	[94]

**Table 2:** Bioactive terpenoids from African flora with potential for antitrypanosomal anti-*Schistosoma* and antileischmanial drug discovery

Compound number	Compound class/subclass	Part of plant studied	Species name	Plant family	Place of collection	Used traditionally/locally	Reported activity on/against	References
**34**–**38**	Terpenoid/Sesquiterpenoids	Stem bark	*Warburgia ugandensis*	Canellaceae	Harena Forest, Dello Menna, Ethiopia	Treatment of various ailments such as common cold, fever, malaria, stomachache, constipation snakebites measles and diarrheal, This plant is also a common component in a number of medicinal preparations.	Trypanosomiasis (IC_50_ from 0.64 to 6.4 µM)	[115]
**39**–**41**	Terpenoid/Carvotacetone derivatives	Aerial parts	*Sphaeranthus bullatus* (syn: *S. gallensis Sacleux*)	Asteraceae	Ngong forest, Nairobi, Kenya	Usually consumed as herbal tea for the management of diarrhea.	Leishmanosomiasis (IC_50_ = 2.16, 10.64 and 2.89 µM, respectively)	[118]
**42**, **43**	Terpenoid	Roots	*Clerodendrum eriophyllum*	Verbenaceae	Machakos, Eastern Kenya	Treatment of malaria	Leishmanosomiasis (IC_50_ = 0.25 and 0.61 µM, respectively)	[121]
**44**–**46**	Terpenoid/Diterpenoid	Leaves	*Polyalthia longifolia*	Annonaceae	Anyigba, Kogi State, Nigeria	To treat various protozoan infections including species of *Trypanosoma*, *Leishmania*, and *Plasmodium*	Trypanosomiasis	[122]
**47**		Leaves	*Eucalyptus maculata*	Myrtaceae	Anyigba, Kogi State, Nigeria	To treat various protozoan infections including species of *Trypanosoma*, *Leishmania*, and *Plasmodium*	Trypanosomiasis	[122]
**48**	Terpenoid/Diterpenoid	Bark	*Entada abyssinica*	Fabaceae	Dschang, Cameroon	To treat sleeping sickness	Trypanosomiasis (IC_50_ = 12 μM)	[124]
**49**–**51**	Terpenoid/Diterpenoid	Fruits	*Xylopia aethiopica*	Annonaceae	Nkongsamba, Cameroon	To treat bronchitis and dysenteric among other ailments	Trypanosomiasis	[126]
**52**	Terpenoid/Diterpenoid	Rhizomes	*Aframomum sceptrum*	Zingiberaceae	Ivory Coast	In addition to their spiritual belief from the plant species, they are as well used as food spice, and for the treatment of inflammation, eczema, fevers, laxative, anti-helmintic, mumps, etc.	Trypanosomiasis and leishmanosomiasis (IC_50_ = 5.7 μM).	[127]
**53**	Terpenoid/Triterpenoid	Roots	*Asparagus stipularis*	Asparagaceae	Sinai, Egypt	To treat Schistosomiasis (bilharziasis) amongst other ailments	Schistosomiasis	[128]
**54**	Terpenoid/Diterpenoid	Root barks	*Elaeodendron schlechteranum*	Celastraceae	Bunda district, Kung’ombe, Tanzania	Treatment of anaemia, general body pain, dysmenorrhea, female infertility and male impotence, boils, carbuncles, cardiovascular problems including hypertension and joint inflammation.	Trypanosomiasis (*T. cruzi* (IC_50_ < 0.57 μM), *T. brucei* (IC_50_ < 0.57 μM) and leishmanosomiasis against (*L. infantum* IC_50_ = 1.67 μM)	[129]
**55**, **56**		Roots	*Salacia madagascariensis*	Celastraceae	Tanzania	Treat malaria, fever, and menorrhagia	Leishmanosomiasis	[130]
**57**, **58**	Terpenoid/Diterpenoid and Triterpenoid	Leaves	*Keetia leucantha* (syn: *Plectronia leucantha Krause*)	Rubiaceae	Benin	To treat parasitic diseases	Trypanosomiasis (IC_50_ for 57 = 5.48 and 14.25 μM, respectively, on *Tbb* BSF and *Tbb* PF. IC_50_ for 58 = 16.00 μM on *Tbb* BSF)	[131]
**59**	Terpenoid/Diterpenoid	Stem bark	*Piptostigma preussi*	Annonaceae	Ebolowa, Cameroon	To treat malaria	Trypanosomiasis activity	[132]
**60**, **61**	Terpenoid/Triterpenoid	Stem bark	*Vernonia guineensis*	Asteraceae (Compositae)	Bafoussam, Cameroon	To treat malaria and jaundice as well as an anthelmintic, an aphrodisiac and an anti-dote to poison	Trypanosomiasis (IC_50_ from 4.60 to 7.67 μM)	[133]

**Table 3:** Other bioactive compounds from African flora with potential for antitrypanosomal and antileischmanial drug discovery

Compound number	Compound class/subclass	Part of plant studied	Species name	Plant family	Place of collection	Used traditionally/locally	Reported activity on/against	RefERENCES
**62**	Amide	Roots	*Zapoteca portoricensis*	Fabaceae	Nsukka, in Enugu State, Nigeria	In wound healing as well as the treatment of toothache, tonsilitis, against diarrhoea, and as an anticonvulsant and antispasmodic	Trypanosomiasis (IC_50_ = 3.63, 41.65 and 92.05 µM against *T. b. rhodesiense*, *T. cruzi* and L6 cells, respectively)	[143]
**63**, **64**	Diarylheptanoid	Seeds	*Aframomum letestuianum*	Zingiberaceae	Abong-bang, Cameroon	In addition to their spiritual belief from the plant species, they are as well used as food spice, and for the treatment of inflammation, eczema, fevers, laxative, anti-helmintic, mumps, etc.	Trypanosomiasis (IC_50_ = 4.49 and 8.39 µM, respectively)	[136]
**65**–**68**	Acylphloroglucinols	Fruits	*Allanblackia monticola*	Clusiaceae (Guttiferae)	Bazou, West Province, Cameroon	Treatment of certain human ailments such as respiratory infections, diarrhoea, toothache, pain, fever	Leishmanosomiasis (IC_50_ = 0.16, 0.33 and 0.2 µM, for 65 to 67, respectively)	[149]
**69**	Xanthone	Leaves	*Symphonia globulifera*	Clusiaceae (Guttiferae)	Bangangté, West Province, Cameroon	To treat malaria, stomach and skin aches. It is also used as laxative by pregnant women and as a general tonic	Leishmanosomiasis	[149]
**70**–**72**	Taccalonolide	Tubers	*Tacca leontopetaloides*	Taccaceae	Benue State, Nigeria	tubers are also processed for food as well as to treat stomach disorders, gastric ulcers, tooth ache, high blood pressure, hepatitis, enteritis and sexual dysfunction	Trypanosomiasis	[150]
**73**–**75**	Quinone/Anthrone	Leaf latex	*Aloe calidophila*	Asphodelaceae	Yabello and Mega, Ethiopia	To treat sexually transmitted infections, digestive disorder, dermatological ailments, opthalmia, conjunctivitis, wounds, burns, other injuries, etc.	Leishmanosomiasis (IC_50_ from 3.12 to 15.26 μM)	[151]
**76**, **77**	Quinone/Naphthoquinone	Seeds	*Triphyophyllum peltatum*	Dioncophyllaceae	Parc de Taï, Ivory Coast	For treatment of malaria, dysentery and elephantiasis	Leishmanosomiasis	[152]
**78**	Lactone	Stems	*Uvaria klainean*	Annonaceae	Forêt des Abeilles, Gabon	For treatment of skin diseases, parasitic infections	Leishmanosomiasis (IC_50_ = 1.75 and 3.12 μM, respectively, against sensitive and amphotericin B-resistant promastigote forms of *L. donovani*)	[153]
**79**	Flavonoid	Leaves	*Vitex simplicifolia*	Verbenaceae	Nsukka, Nigeria	To treat edema, gout, malaria, skin diseases, toothache and dermatitis	Trypanosomiasis (IC_50_ = 4.7 μg/mL)	[154]
**80**		Aerial parts	*Ageratum conyzoides*	Asteraceae (Compositae)	Nile bank, Khartoum, Sudan	To treat leprosy, skin diseases, wound healing, mental headaches, dyspnea and infectious diseases. It is also used locally for its anti-asthmatic, antispasmodic, haemostatic effects and as an oil lotion for purulent ophthalmia.	Trypanosomiasis (IC_50_ = 7.8 μM) and leishmanosomiasis (IC_50_ = 9.2 μM)	[155]
**81**, **82**	Phytosterol	Stem bark	*Allexis cauliflora*	Violaceae	Ebolowa, Cameroon	To treat fever and syphilis	Trypanosomiasis	[156]

## Alkaloids

**Fig. 2:**
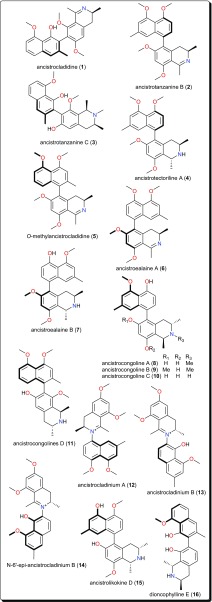
Antiprotozoal naphthylisoquinoline alkaloids

**Fig. 3:**
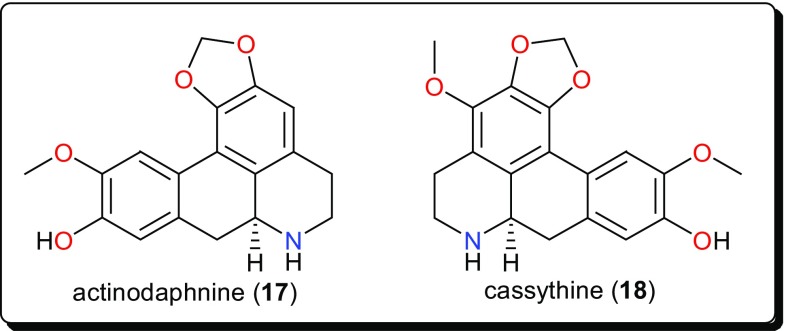
Aporphine alkaloids with trypanosidal potencies

**Fig. 4:**
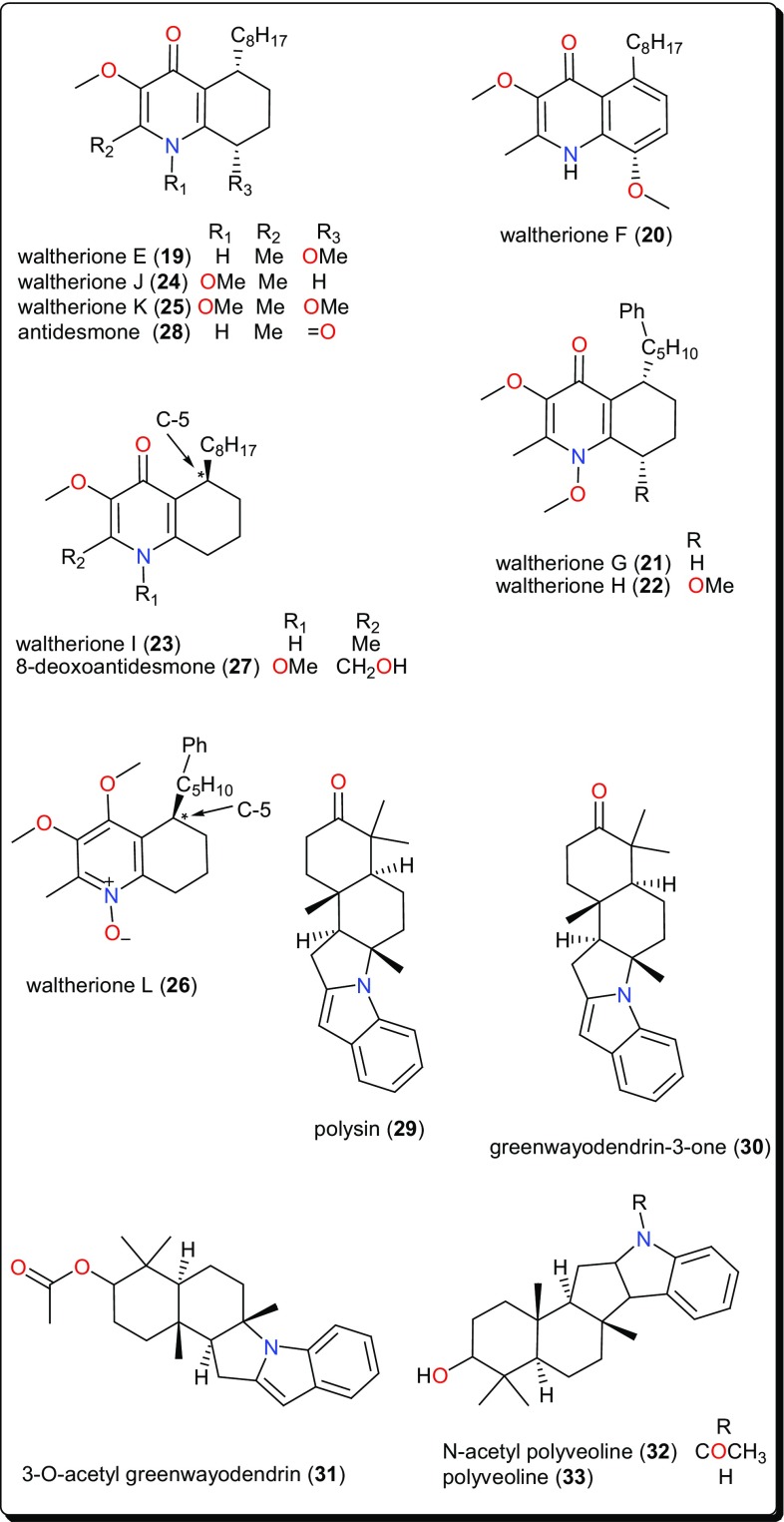
Quinoline, indoles and other alkaloids showing activities against *Trypanosoma* species

**Fig. 5:**
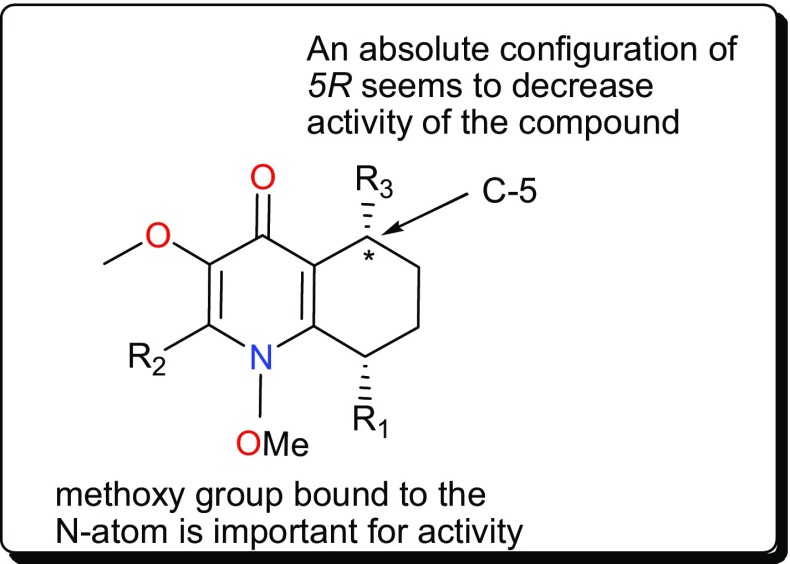
SAR for *W. indica* compounds inhibiting *T. cruzi*, *T. b. brucei* and *T. b. rhodesiense*

This class is characterized by nitrogen-containing compounds that are naturally occurring. Diverse species (fungi, plants, animals) have yielded several bioactive alkaloids against a broad range of diseases [76–84]. Table 1 summarises the alkaloids (compounds **1**–**33**) isolated from AMPs and evaluated against these parasitic diseases, while Figs. 2, 3, 4 and 5 show a selection of some promising alkaloidal compounds, based on their evaluated activity (< 12.41 µM).

### Naphthylisoquinolines

The leaves, stem bark and roots of *Ancistrocladus* sp. (Ancistrocladaceae) are known to be rich sources of naphthylisoquinoline alkaloids (Fig. 2) [85–91]. Ancistrocladidine (**1**), ancistrotanzanines B (**2**), and C (**3**), ancistrotectoriline A (**4**), *O*-methylancistrocladidine (**5**), ancistroealaines A (**6**) and B (**7**), ancistrocongolines A-D (**8**–**11**), ancistrocladiniums A (**12**) and B (13), *N*-6′-epi-ancistrocladinium B (**14**), ancistrolikokine D (**15**) and dioncophylline E (**16**) are few examples of naphthylisoquinoline antiparasitic alkaloids from *Ancistrocladus* sp. and *Dioncophyllum thollonii* (Dioncophyllaceae).

The evaluation of the biological activities of these compounds showed them to be a rare set and promising class of antiprotozoal and antiviral agents, which are only found in plants of the Ancistrocladaceae and Dioncophyllaceae, mostly found in Africa. Their anti-*Trypanosoma* activities are evident (e.g., with IC_50_ values ranging from 0.17 to 12.41 µM against *Trypanosoma brucei rhodesiense*), alongside good to moderate activities against *Trypanosoma cruzi* and *Leishmania donovani.* It might be worth mentioning that the isoquinoline scaffold has also been explored synthetically for the discovery of novel antiprotozoals and antimicrobials [85, 86, 95–97].

### Aporphines

Other bioactive alkaloids include the aporphines (Fig. 3); actinodaphnine (**17**) and cassythine (**18**) from *Cassytha filiformis* (Lauraceae), a plant whose alkaloidal extract showed activity against *T. b. brucei* (with an IC_50_ value of 2.2 μg/mL). This confirmed the use of this plant in African folkloric medicine to treat African Trypanosomiasis and other diseases [92, 98, 99]. The isolated compounds displayed antitrypanosomal activities, with IC_50_ values of 10.29 and 17.60 μM for compounds **17** and **18**, respectively. Although, the compounds showed low selectivity indices to HeLa cells (e.g., for actinodaphnine, IC_50_ (HeLa)/IC_50_ (*T. b. brucei*) < 5), when compared with the alkaloidal fraction (selectivity index = 16), they represent good starting scaffolds that could be optimised in order to improve the efficacy and selectivity in the search for new bioactive molecules with trypanocidal effects.

### Quinolines

Other trypanocidal alkaloids include the quinolines (Fig. 4); waltheriones E–L (**19**–**26**), 8-deoxoantidesmone (**27**) and antidesmone (**28**) from *Waltheria indica* (Malvaceae) [93]. This plant is used in traditional medicine for the treatment of several ailments, including malaria [63, 100–104]. The dichloromethane root extract showed activities against *T. cruzi* (IC_50_ = 0.74 μg/mL), *T. b. brucei* (2.3% survival at 20 μg/mL) and *T. b. rhodesiense* (IC_50_ = 17.4 μg/mL) [93]. With the exception of waltherione L (**26**), with a slightly higher IC_50_ (3.1 μM), the isolated compounds all displayed potent growth inhibition toward the amastigote form of *T. cruzi* (the Tulahuen C4 strain), with IC_50_ values lower than that of the reference drug benznidazole (IC_50_ = 2.9 μM). Structure–activity relationships (SARs) provide suggestions that, a methoxy group, bound to the nitrogen atom is important for activity (e.g., as in compounds **22**, **24** and **25**). This group at this position increased the lethality of *T. cruzi*. Furthermore, the absolute configuration (*5R*) (as in compounds **23**, **26**, **27**) seems to result in a decrease of activity, while the presence of an N-oxide function (as in compound **26**) is detrimental for *T. cruzi* inhibitory activity (Fig. 5). Finally, a comparison of the IC_50_ values of the isolated compounds against *T. brucei* sp. and *T. cruzi* highlighted selective toxicity towards the latter. This suggests that these molecules (or the waltherione scaffold) is a potential starting point for new safe antitrypanocidal drug development, although antidesmone (**28**) has already been patented for its potential as an antiprotozoal drug since 2003 [93, 105, 106].

### Indoles and Other Alkaloids

Polysin (**29**), an indolosesquiterpene alkaloid from *Polyalthia suaveolens* (Annonaceae), was isolated together with the known alkaloids (Fig. 4); greenwayodendrin-3-one (**30**), 3-*O*-acetyl greenwayodendrin (**31**), *N*-acetyl polyveoline (**32**) and polyveoline (**33**). These alkaloids have demonstrated interesting activities on selected glycolytic enzymes, e.g., phosphofructo kinase (PFK), glyceraldehyde-3-phosphate dehydrogenase (GAPDH) and aldolase [94]. Of particular interest are polysin (**29**) and 3-*O*-acetyl greenwayodendrin (**31**). Compound **29** acted as a competitive reversible inhibitor against *T. brucei* PFK (*K*_*i*_ = 10 μM), while compound** 31** acted as a selective inhibitor of *T. brucei* aldolase (with IC_50_ ~ 0.5 μM). Meanwhile, polyveoline (**33**) acted as a selective inhibitor of *T. brucei* PFK and is a mixed reversible inhibitor of *T. brucei* GAPDH. These compounds, therefore, represent a good starting point for the design of new selective and potent trypanosomal drugs.

## Terpenoids

**Fig. 6:**
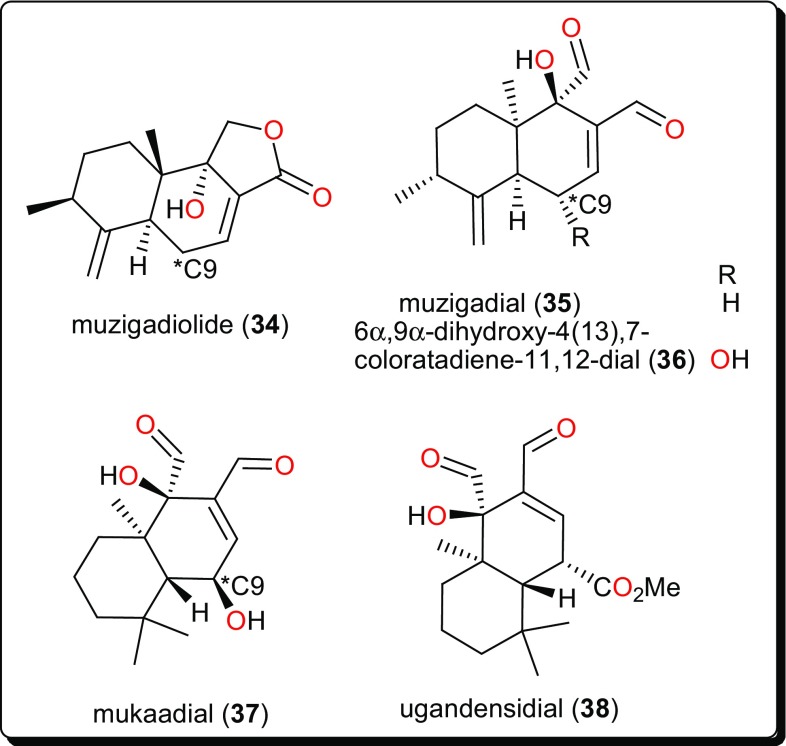
Sesquiterpenoids which have demonstrated anti-*Trypanosoma* activities

**Fig. 7:**
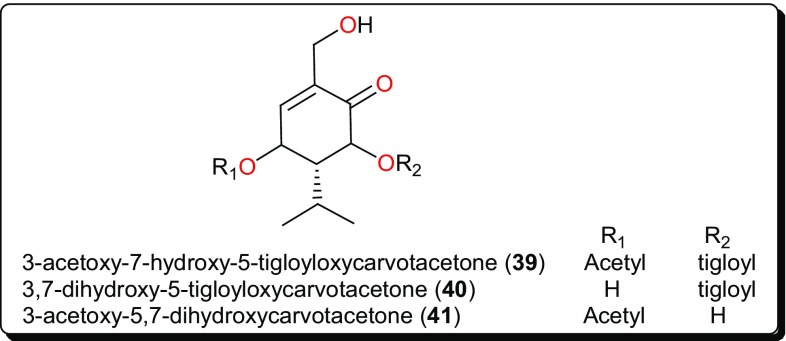
Carvotacetones with potent antileishmanial activities

**Fig. 8:**
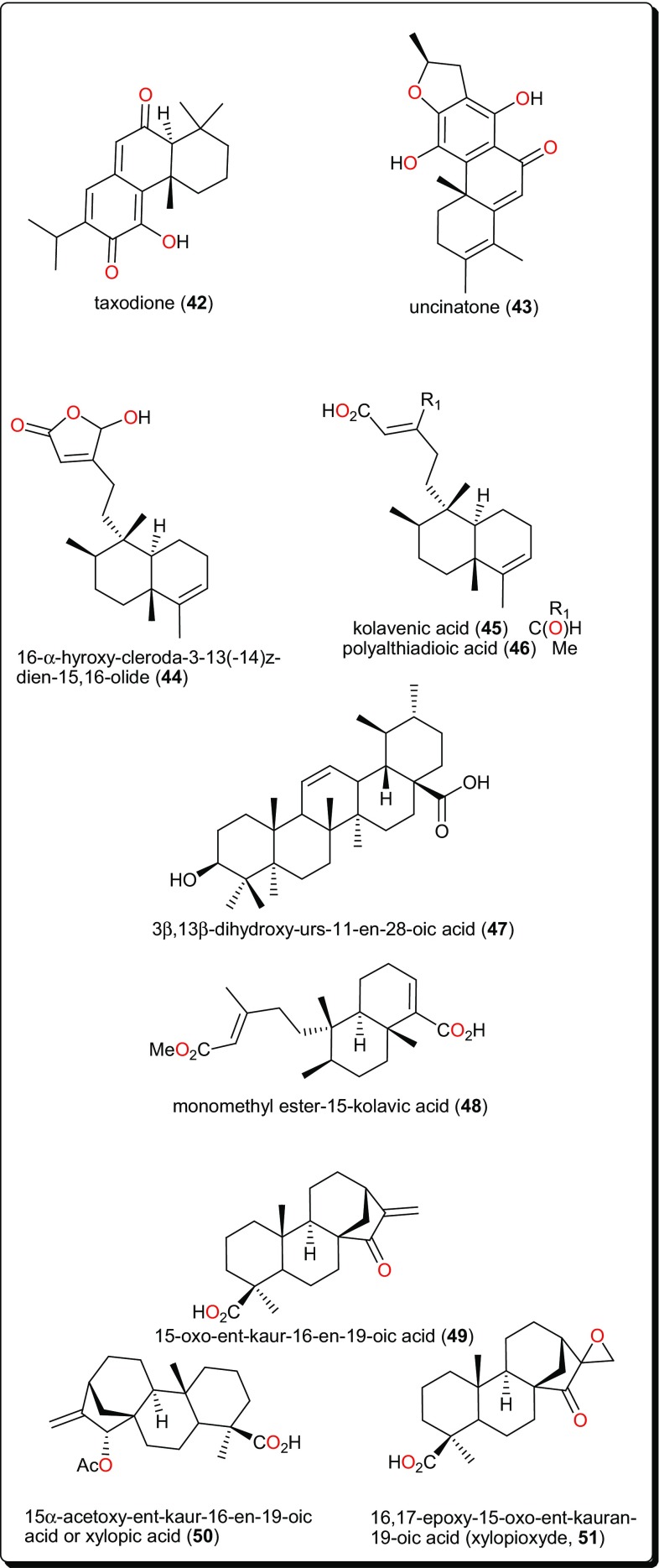
Diterpenoids and a triterpenoid with selective inhibitory activity against *T. brucei* GAPDH

**Fig. 9:**
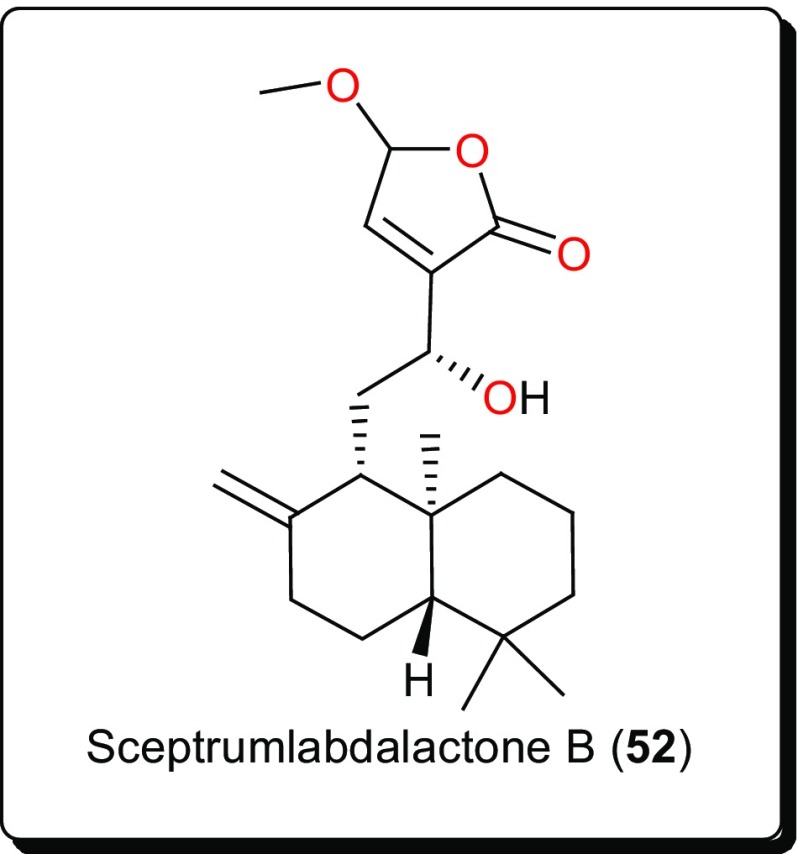
Potent compound with selective activity for *L. donovani*, when compared with the activity against *T. b. brucei*

**Fig. 10:**
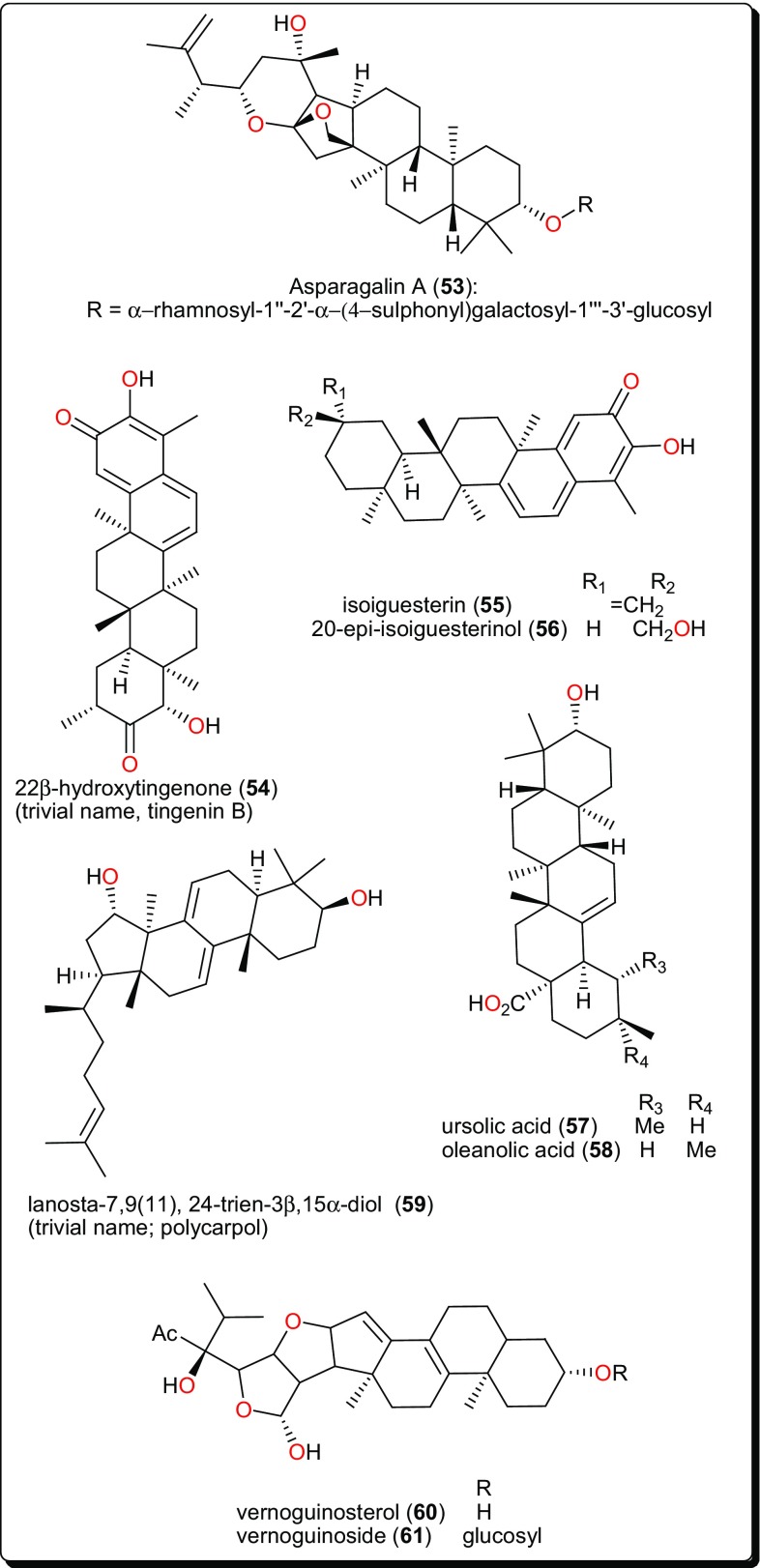
Triterpenoids with antiparasitic activities

Terpenoids constitute a large and diverse class of naturally occurring secondary metabolites, with interesting physiological and pharmacological functions [44, 107–110]. Their main scaffolds occur as multicyclic structures, e.g., hemi-terpenoids (5 carbon atoms), monoterpenoids (10 carbon atoms), sesquiterpenoids (15 carbon atoms), diterpenoids (20 carbon atoms), sesterterpenoids (25 carbon atoms), triterpenoids (30 carbon atoms), tetraterpenoids (40 carbon atoms), and polyterpenoids (more than 40 carbon atoms), which are all primarily derived from the five-carbon isoprene units [45, 107]. Terpenoids have been proven to possess interesting pharmacological activities as seen in the summary presented in Table 2 (compounds **34**–**61**) and their corresponding structures shown in Figs. 6, 7, 8, 9 and 10 [44, 111–114].

### Sesquiterpenoids

The sesquiterpenoids (Fig. 6), muzigadiolide (**34**), muzigadial (**35**), 6α,9α-dihydroxy-4(13),7-coloratadiene-11,12-dial (**36**), mukaadial (**37**) and ugandensidial (**38**), from the East African medicinal plant *Warburgia ugandensis* (Canellaceae) have demonstrated anti-*Trypanosoma* activities [115]. The compounds displayed in vitro activities (with IC_50_ values ranging from 0.64 to 6.4 µM) against *T. b. rhodesiense*, the parasite responsible for African sleeping sickness. Compound** 37** had previously been isolated from the same plant, also showing antitrypanocidal activity [116]. This plant (now regarded as an endangered species) has attracted many researchers because of its traditional use for the treatment of a variety of ailments, including malaria and diverse fevers [115–117]. SAR studies suggested that an additional dialdehyde functional group to the sesquiterpene lactone backbone, together with a hydroxyl group attached to C-9 contribute to the activity of the compounds.

### Carvotacetone Derivatives

The native tropical East African medicinal plant *Sphaeranthus bullatus* (synonym: *S. gallensis* Sacleux, Family: Asteraceae) has been the origin of several compounds (Fig. 7) [118–120], including the carvotacetone derivatives; 3-acetoxy-7-hydroxy-5-tigloyloxycarvotacetone (**39**), 3,7-dihydroxy-5-tigloyloxycarvotacetone (**40**) and 3-acetoxy-5,7-dihydroxycarvotacetone (**41**). Compounds **39**–**41** demonstrated antileishmanial activities, with IC_50_ values of 2.16, 10.64 and 2.89 µM, respectively, against the parasite *L. donovanii* promastigotes.

### Diterpenoids

Other terpenoids include the abietane diterpenoids, taxodione (**42**) and uncinatone (**43**), Fig. 8, from the roots of *Clerodendrum eriophyllum* (Verbenaceae) [121], which displayed potent antileishmanial activities (with IC_50_ values of 0.25 and 0.61 µM, respectively) against *L. donovanii*. The activities of the crude extracts, e.g., the ethyl acetate extracts of *Newbouldia laevis* (Bignoniaceae) (EC_50_ 4.2 µg/mL) and *Eucalyptus maculata* (Myrtaceae) (EC_50_ 12.3 μg/mL) and the hexane extract of *Polyalthia longifolia* (Annonaceae) (EC_50_ 2.4 µg/mL) as well as their isolated active compounds (Fig. 8); 16-α-hydroxy-cleroda-3-13(-14)z-dien-15,16-olide (**44**), kolavenic acid (**45**), polyalthiadioic acid (**46**) and the triterpenoid 3β,13β-dihydroxy-urs-11-en-28-oic acid (**47**) were observed against different trypanosomes strains (s427 WT, B48 and AQP2/3KO) [122]. While these pure compounds exhibited activities against the tested strains, with EC_50_ values ranging from 1.16 to 40.46 μM, it was remarkable that no toxicity towards Human Embryonic Kidney cells was observed even at concentrations up to 400 µg/mL (1.31 μM), thus suggesting new scaffolds to be further developed for the treatment of the wild-type and multi-drug resistant *T. brucei* [122, 123]. Also interesting is the kolavic acid derivative; monomethyl ester-15-kolavic acid (**48**) isolated from *Entada abyssinica* (Fabaceae) [124], which demonstrated interesting selective inhibitory activity (IC_50_ value of 12 μM) against *T. brucei* GAPDH [125].

Other bioactive diterpenoids include 15-oxo-*ent*-kaur-16-en-19-oic acid (**49**), 15α-acetoxy-*ent*-kaur-16-en-19-oic acid or xylopic acid, (**50**) and 16,17-epoxy-15-oxo-*ent*-kauran-19-oic acid or xylopioxyde (**51**), from the fruits of *Xylopia aethiopica* (Annonaceae) [126]. These compounds and their synthetic epoxide analogues were screened on antitrypanosomal and cytotoxicity assays, showing that only the naturally-occurring compounds (**49**–**51**) displayed cytotoxic effects on the mammalian fibroblast cell line MRC-5 (with ED_50_ values ranging from 22 to 121 µM), as well as inhibitory effects on the growth of the bloodstream forms of *T. b. brucei* cells (strain 241) (ED_50_ ranging from 27 to 205 µM).

The genus *Aframomum* (Zingiberaceae), has been the source of the antitryposonomals. Sceptrumlabdalactone B (**52**, Fig. 9) was identified, from the rhizomes of *A. sceptrum*, a plant locally used for the treatment of infectious diseases including human African Trypanosomiasis (sleeping sickness), together with sceptrumlabdalactone A [127, 134–136]. The activity of compound **52** (with IC_50_ value of 5.7 μM) against *L. donovani* was comparable to that of reference drugs (IC_50_ of 2.5 and 3.0 μM for pentamidine and miltefosine respectively). Additionally, this molecule demonstrated selective activity for *L. donovani*, when compared with the activity against *T. b. brucei*.

### Triterpenoids

The anti-schistosomal activity of Asparagalin A (**53**, Fig. 10), from the Egyptian medicinal plant *Asparagus stipularis* (Asparagaceae) has been evaluated [128]. It was found that this compound was able to significantly reduce the ability of adult female worms to lay eggs. It was further shown that the compound had some suppressive effect on egg-laying capacity in a dose-dependence manner [137]. *Elaeodendron schlechteranum* (Celastraceae) is the source of tingenin B or 22β-hydroxytingenone (**54**) [129]. This compound has displayed a broad range of activities, e.g., against *T. cruzi* (IC_50_ < 0.57 μM), *T. brucei* (< 0.57 μM), *L. infantum* (1.67 μM), and *P. falciparum* (0.83 μM), confirming the claim of the applicability of the plant in traditional medicine to treat various non-infectious diseases [63, 138]. Albeit, being highly cytotoxic to MRC-5 cells (CC_50_ 0.45 μg/mL), compound** 54** indicates a poor selectivity to normal cells. Further studies on this compound could be considered in order to suggest less toxic and more selective analogues for the development of novel antiparasitics. The bisnortriterpenes from *Salacia madagascariensis* (Celastraceae); isoiguesterin (**55**) and 20-*epi*-isoiguesterinol (**56**) showed potent activities against *Leishmania* sp. [130]. Meanwhile, isoiguesterin (**55**) and 20-*epi*-isoiguesterinol (**56**) displayed comparable activities with chloroquine and artemisinin against the D6 clone, being more potent and selective against *L. donovani* (a species known to cause visceral Leishmaniasis). When compared with amphotericin B, used currently in the treatment of Leishmaniasis, compounds **55** and **56** show great potential for future selective drug development against *Leishmania*.

*Keetia leucantha* (synonym: *Plectronia leucantha* Krause) is a West African tree of the Rubiaceae, used to treat a variety of infections, including parasitic infections [139, 140]. Ursolic acid (**57**) and oleanolic acid (**58**), along with other constituents were isolated from the leaves of this plant. An investigation of the antitrypanosomal activities of essential oil, the dichloromethane extract and isolated compounds on *T. b. brucei* bloodstream forms (*Tbb* BSF) and procyclic forms (*Tbb* PF) [131] showed that ursolic acid (**57**) and oleanolic acid (**58**) were the most bioactive tested compounds [131]. Ursolic acid displayed IC_50_ values of 5.48 and 14.25 μM, respectively, on *Tbb* BSF and *Tbb* PF, while oleanolic acid displayed an IC_50_ value of 16.00 μM on *Tbb* BSF. This could explain why the plant is effective in the traditional treatment of related parasitic ailments. Another identified triterpenoid was polycarpol or lanosta-7,9(11),24-trien-3β,15α-diol (**59**) from *Piptostigma preussi* (Annonaceae) [132]. The compound showed antitrypanosomal activity with an ED_50_ value of 5.11 µM on *T. brucei* cells. An investigation of its mode of action showed that the compound acted by inhibiting *T. brucei* glycolytic enzymes GAPDH and PFK (glycolytic pathway enzymes validated by WHO as good targets for the development of drugs against trypanosomiasis), with IC_50_ values of 650 and 180 µM, respectively. The glycolytic enzymes GAPDH are responsible for ATP production and have been reported to be vital for the survival of Trypanosomatids [141, 142]. From the stem bark of *Vernonia guineensis* (Asteraceae), vernoguinosterol (**60**) and vernoguinoside (**61**), exhibited interesting trypanocidal activity with IC_50_ values in the range 4.60–7.67 μM [133].

## Other Compound Classes

**Fig. 11:**
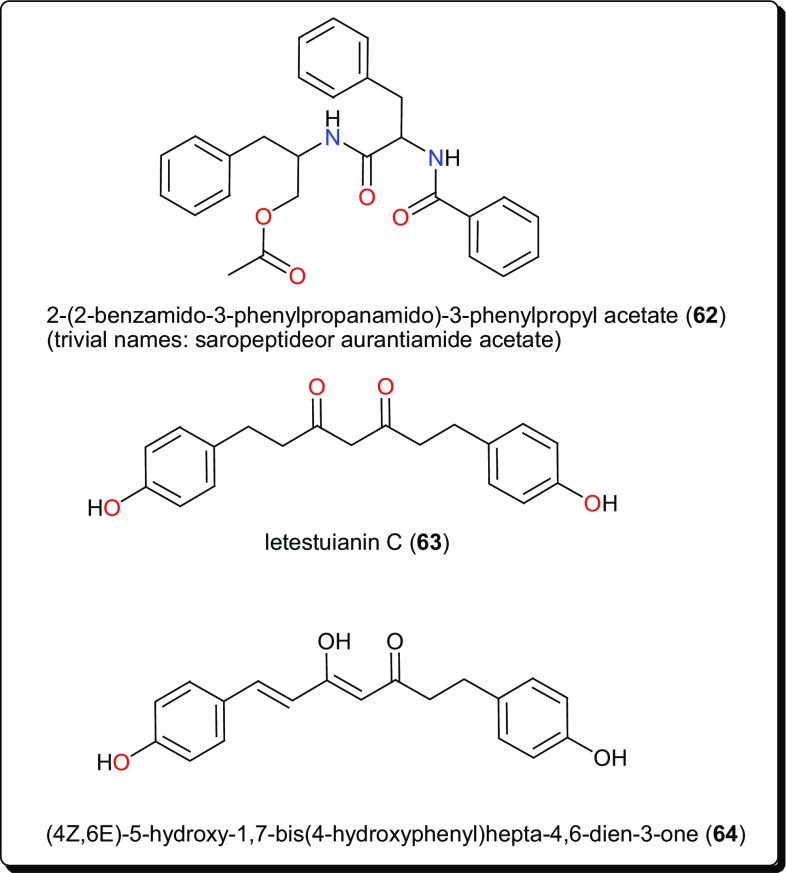
An antitrypansomal amide and two diarylhepanoids

**Fig. 12:**
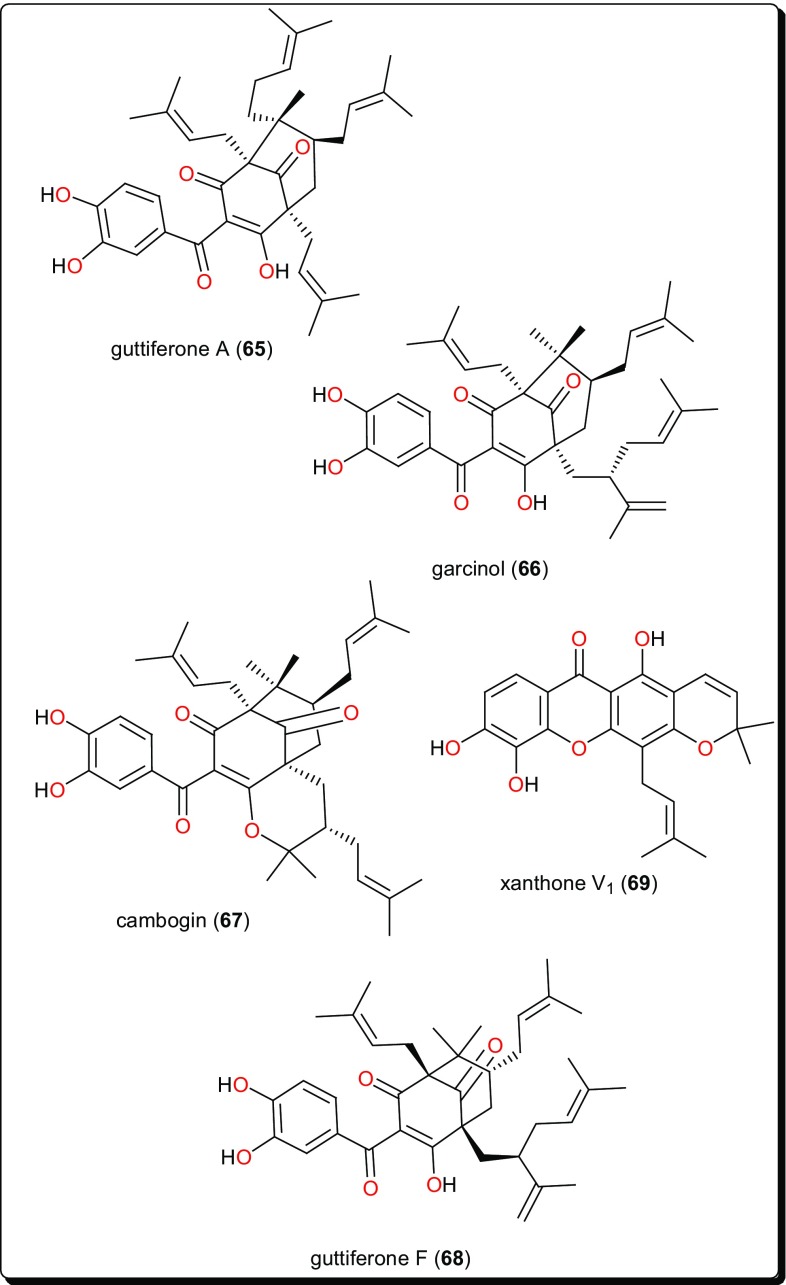
Acylphloroglucinols and a xanthone with very potent in vitro antileishmanial activities in the nanomolar range

**Fig. 13:**
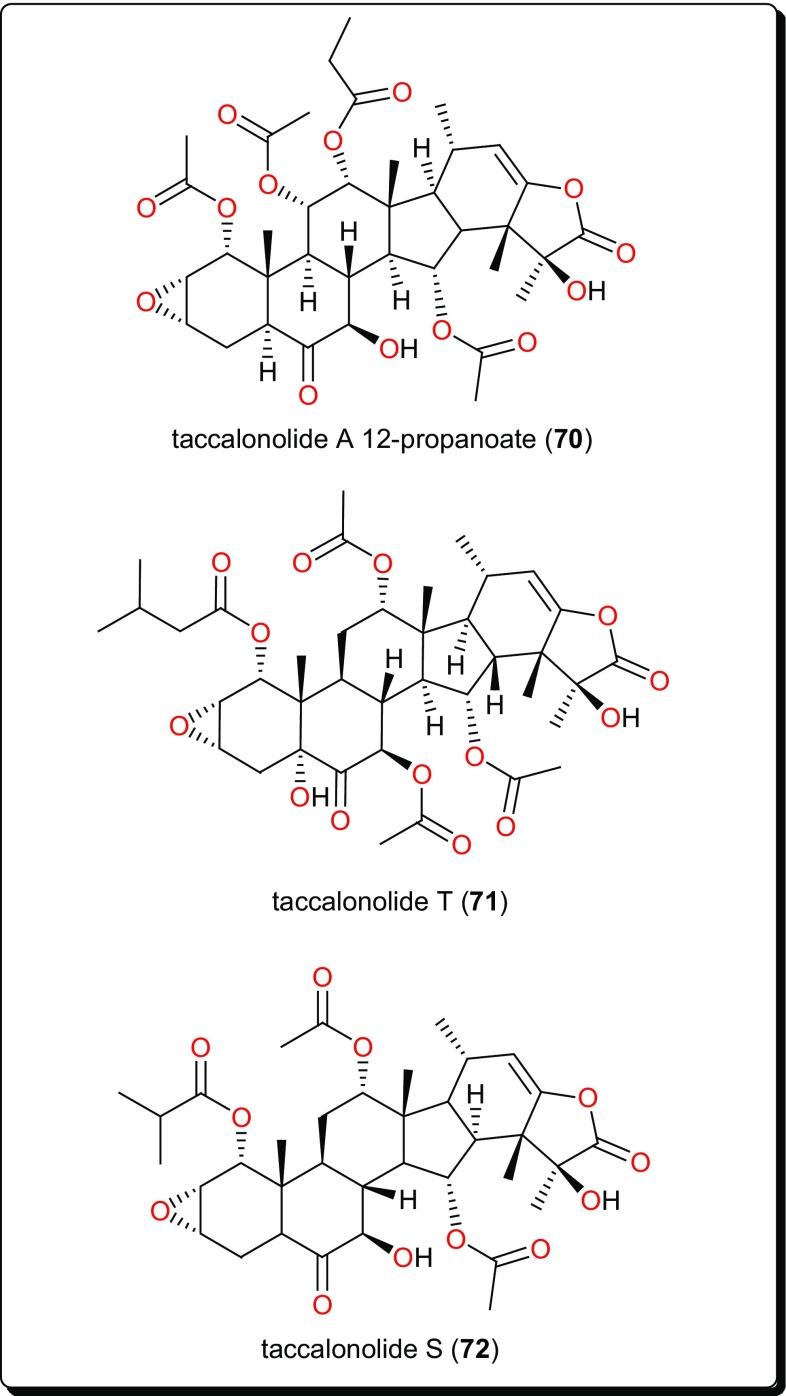
Taccalonolides, a rare class of antiprotozoals

**Fig. 14:**
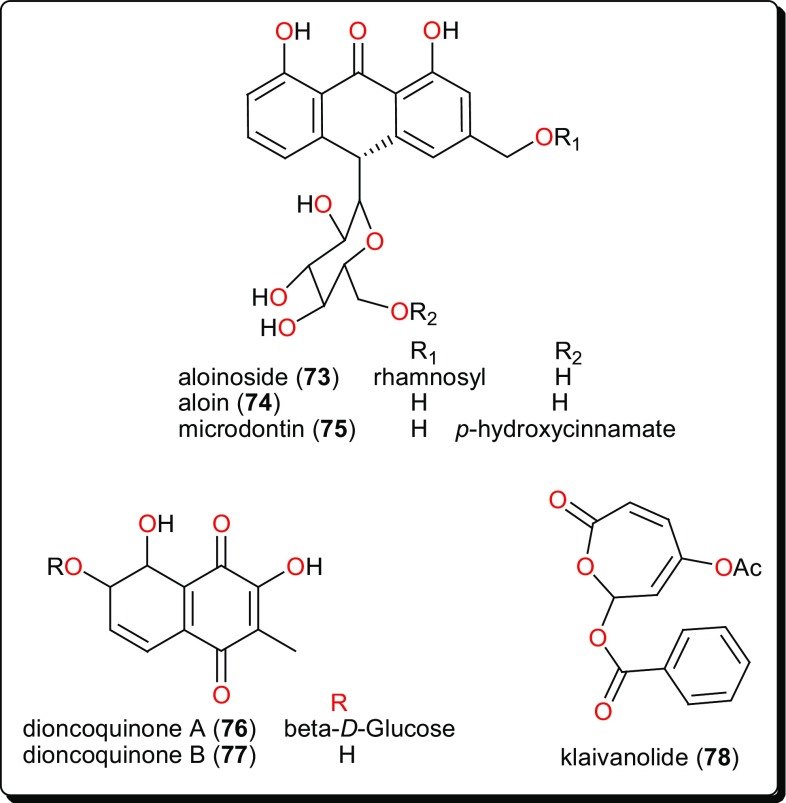
Quiniones and klaivanolide, which showed lower micromolar activities against against several *Leishmania* species

**Fig. 15:**
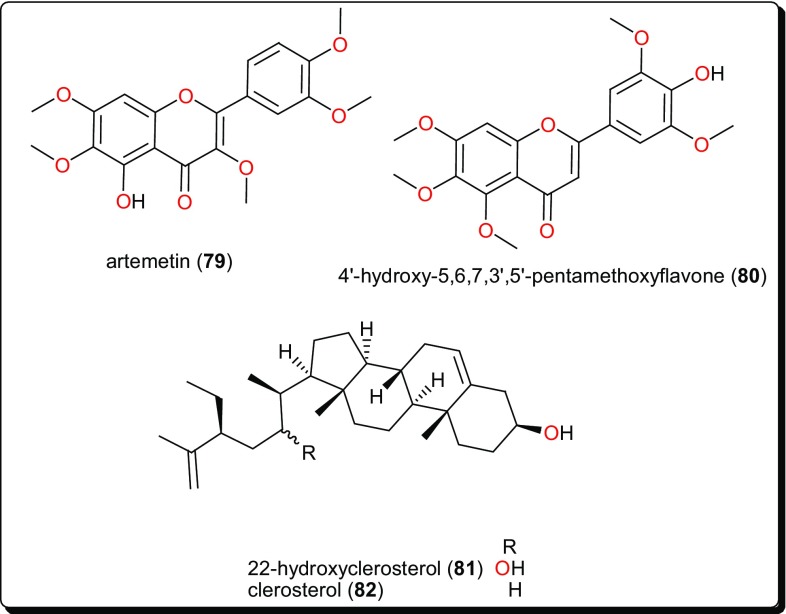
Flavonoids and phytosterols with trypanocidal activities

Other compound classes from AMP with reported activities on Leishmaniasis and Trypanosomiasis are shown in Figs. 11, 12, 13, 14, and 15, while a summary of the reported molecules is given in Table 3 (compounds **62**–**82**).

### Amides

Plants from the genus *Zapoteca* (Fabaceae) have been the origin of diverse compounds with antiprotozoal activities. These include the ester 2-(2-benzamido-3-phenylpropanamido)-3-phenylpropyl acetate (trivial names; saropeptide or aurantiamide acetate) (**62**) from *Z. portoricensis* (Fig. 11) [143]. The IC_50_ values of compound **62** were 3.63, 41.65 and 92.05 µM against *T. b. rhodesiense*, *T. cruzi* and against rat skeletal myoblast cell line (L6 cells), respectively. The compound had been previously reported to possess anti-inflammatory as well as antiplatelet aggregation activities, which are complementary to the observed trypanocidal property [144–148]. Since inflammation poses major problems in the advanced stages of Trypanosomiasis, compound **62** represents a promising natural hit with a reasonable selectivity for *T. b. rhodesiense*.

### Diarylhepanoids

Other potent antitrypanosomal compounds are the diarylheptanoid; letestuianin C (**63**) and (4*Z*,6*E*)-5-hydroxy-1,7-bis(4-hydroxyphenyl)hepta-4,6-dien-3-one (**64**) from the species *Aframomum letestuianum*, Fig. 11 [136]. The activities of compounds 63 (4.49 µM) and 64 (8.39 µM) validate the use of the *Aframomum* sp. in treating parasitic ailments amongst others.

### Acylphloroglucinols and Xanthones

Guttiferone A (**65**), garcinol (**66**), cambogin (**67**) and guttiferone F (**68**) from *Allanblackia monticola* (Guttiferae or Clusiaceae) fruits and xanthone V_1_ (**69**) from *Symphonia globulifera* leaves have shown antileishmanial activities (Fig. 12) [149]. The compounds exhibited very potent in vitro antileishmanial activities, particularly compounds **65**–**67**, with IC_50_ values of 0.16, 0.33 and 0.2 µM, for compounds **65**, **66** and **67**, respectively. These were lower than that of the reference drug, miltefosine (0.46 µM). SAR studies could further improve the activities of these compounds in order to enhance their selectivity indices against human cancer cell lines.

### Taccalonolides

Beside their proven anticancer potential, these represent a quite potent class of antitrypanosomal compounds identified from *Tacca leontopetaloides* (Taccaceae) [157–162]. These include taccalonolide A 12-propanoate (**70**), taccalonolide T (**71**) and taccalonolide S (**72**) from the tubers of *T. leontopetaloide* (Fig. 13). They have shown activities against the *T. b. brucei* s427 lister strain [150]. These compounds and crude fractions yielded EC_50_ values as low as 0.79 μg/mL.

### Quiniones and Klaivanolide

Quinones from *Aloe* species have also shown antileishmanial activities. These include aloinoside (**73**), aloin (**74**) and microdontin (**75**) from the leaf latex of *A. calidophila* (Fig. 14) [151]. It is noteworthy that, the activities of the most potent compounds, with IC_50_ values ranging from 3.12 to 10.92 μM against *Leishmania aethiopica* and from 3.70 to 15.26 μM against *Leishmania major*, were comparable to the control drug amphotericin B (IC_50_ = 0.12 and 0.07 μM against *L. aethiopica* and *L. major* respectively). The selectivity indices of aloinoside (**73**) (813.35 and 694.90, respectively, against *L. aethiopica* and *L. major*) were much better than those of the control, amphotericin B (423.49 and 688.96, respectively). This suggests that the isolated compounds could serve as potential scaffolds for the development of safe, specific and cost-effective antileishmanial agents [151]. Additionally, the dioncoquinones A (**76**) and B (**77**) isolated from *Triphyophyllum peltatum* (Dioncophyllaceae) showed good and specific activity against *L. major* by inhibiting the growth of the parasite at very low concentrations [152]. Klaivanolide (**78**), from the stems of *Uvaria klaineana* (Annonaceae), was also reported as a potent molecule (in vitro IC_50_ values of 1.75 and 3.12 μM, respectively) against sensitive and amphotericin B-resistant promastigote forms of *L. donovani* [153].

### Flavonoids

Artemetin (**79**, Fig. 15), from *Vitex simplicifolia* (Verbenaceae) leaves, exhibited promising trypanocidal activity with an IC_50_ value of 4.7 μg/mL and a selectivity index of 9.8 against L6 cells [154]. While this activity confirms the use of this plant in the traditional treatment of ailments including parasitic diseases [163–165], phytochemical evaluation of trypanocidal activities were not reported before. Hence, the plant could further be investigated for the unidentified compounds. An investigation of *Ageratum conyzoides* (Asteraceae), a plant known for its importance in the treatment of sleeping sickness patients traditionally [155, 166, 167], led to the isolation of several flavonoids; 5,6,7,8,5′-pentamethoxy-3′,4′-methylenedioxyflavone (trivial name: eupalestin), 5,6,7,5′-tetramethoxy-3′,4′-methylenedioxyflavone, 5,6,7,8,3′,4′,5′-heptamethoxy-flavone (trivial name: 5′-methoxynobiletine), 5,6,7,3′,4′,5′-hexamethoxy-flavone and 4′-hydroxy-5,6,7,3′,5′-pentamethoxyflavone (trivial name: ageconyflavone C, **80**) which displayed antiprotozoal activities, some in the lower micromolar range [155]. Among the tested NPs, compound **80** showed the highest activity against *T. b. rhodesiense* and *L. donovani* with IC_50_ values of 7.8 and 9.2 μM respectively. However, all the isolated compounds showed an activity weaker than that of the crude extract, implying that the activities of the compounds in the mixture could be synergistic.

### Phytosterols

22-Hydroxyclerosterol (**81**) and clerosterol (**82**), Fig. 15, were isolated from the stem bark of *Allexis cauliflora* (Violaceae) [155]. These compounds were evaluated for trypanocidal activities, and the activity of compound **81** (ED_50_ = 1.12 µM) was far better than that of compound **82** (ED_50_ = 134.34 µM). These results prompted an investigation of their cytotoxic activities. It was observed that compound **81** inhibited mammalian cells at quite a similar concentration (ED_50_ = 1.56 µM), while compound **82** had no effect. This difference in activity could be attributed to the presence of the hydroxyl group at C-22 in the side chain of compound **81** which is absent in compound **82**. Additionally, it was observed that compound **81** was more active and selective on the parasite enzyme glycolytic enzymes (PGI and GAPDH), when compared with compound **82**.

## Conclusions

Parasitic diseases continue to represent a menace on a global scale and require attention due to lack of vaccines and reported resistance against available drugs for their treatment. This review focuses on different natural compounds and scaffolds that could lead drug discovery research groups into reasonable starting points for further development of fast, effective and affordable novel molecules for the treatment of parasitic diseases. Drug discovery and development now place efforts on the search for new moieties or chemical scaffolds of natural/semisynthetic origin and in the development of phytomedicines. As a means to facilitate accessibility of information, our research team has as one of its goals, to develop free online natural products libraries from African flora (http://african-compounds.org/). In this paper, an attempt has been made to draw together original research works on natural products from AMP with micromolar range activities against *Schistosoma*, *Trypanosoma* and *Leishmania* species. The compounds presented herein have demonstrated a diverse range of activities against different forms of Trypanosomiasis, Schistosomiasis and Leishmaniasis, with some scaffolds and molecules showing great potential as starting points for further development into drugs. We recently collected a dataset of several hundred bioactive plant based metabolites from AMPs with activities against *Trypanosoma* sp. (Afrotryp) [68]. It becomes interesting to perform in silico prediction of binding modes and binding free energy calculations of some of the compounds against some selected targets.
